# Determining household out of pocket payments, incidence of catastrophic expenditures and impoverishment among patients with malaria in Zambia’s path towards Universal Health Coverage

**DOI:** 10.1371/journal.pone.0312906

**Published:** 2024-12-10

**Authors:** Patrick Banda, Felix Masiye, Oliver Kaonga, Jesse Bump, Peter Berman

**Affiliations:** 1 Ministry of Health, Lusaka, Zambia; 2 Department of Economics, School of Humanities and Social Sciences, University of Zambia, Lusaka, Zambia; 3 Harvard T. Chan School of Public Health, Harvard University, Cambridge, Massachusetts, United States of America; 4 School of Public Health and Population, University of British Columbia, Vancouver, British Columbia, Canada; PLOS: Public Library of Science, UNITED KINGDOM OF GREAT BRITAIN AND NORTHERN IRELAND

## Abstract

**Background:**

The World Health Organisation (WHO) estimates that about 3.2 billion people which is nearly half of the world’s population are at risk of malaria. Annually about 216 million cases and 445,000 deaths of malaria occur globally. Africa accounted for 90% and 91% of the malaria cases and deaths respectively. Zambia has earmarked malaria elimination on its path to Universal Health Coverage (UHC). This paper aims to determine the incidence of Out-of-Pocket Payments (OOP) and Catastrophic Health Expenditures (CHE) and impoverishment among households with malaria patients in Zambia. The paper focusses on the incidence of OOP and impoverishment for malaria in a setting without user fees for accessing primary malaria health care services and virtually no user fees at all levels of care if referred through the referral system. The results of this study will also serve as a baseline for tracking Zambia’s path towards achieving malaria financial access on its path towards UHC among patient with malaria.

**Methods:**

The study uses a nationally representative cross-sectional survey of households in both rural and urban areas of Zambia. The study employed probability sampling procedures. A two-stage stratified cluster sample design was used. We analyse a total of 2,005 households that had at least one member suffering from malaria with a recall period of four weeks for out-patients and six months for the in-patient respectively. A logistic regression model was estimated with a Categorical Dependent variable being CHE (CHE = = 1, or otherwise = = 0). A household is considered impoverished if it fell below the poverty line due to OOP. All data was analyzed using Stata version 2013.

**Results and discussion:**

The results show that although the country has a free malaria policy at primary care level and virtually at all levels if referred through the health system process, households are still incurring costs in accessing health care services. Incidence of CHE and impoverishment were reflected at all levels. In terms of CHE, the poorest contributed almost 30% while the wealthier quintile contributed about 10%. Similarly, impoverishment effects of OOPs are more pronounced in the poorest quintile. The OOP composed mainly of transport, followed by diagnosis and medicines and was lowest for Insecticide-treated bed nets (ITNs) payments. The high costs of transport that the households had to incur when accessing health services could be due to the long distance that the households have to face as they travel to the health facilities as most of the facilities in Zambia are still outside the 5 km radius. The drug expenditure could be explained by the drugs running out of stock. Low expenditure on ITNs could be due to the country’s strategy of mass distribution working to give the country’s universal financial protection on ITNs for malaria.

**Conclusion and policy implications:**

This study sought to address gaps in OOP and the associated incidence of CHE and impoverishment for malaria, distribution of OOP among Social Economic Status (SES) setting and determinants of OOP in Country that has earmarked malaria elimination in the UHC agenda. Understanding household’s costs related to malaria will enable targeting intervention to accelerate Zambia’s path towards elimination of malaria and therefore contribute to attainment of the Sustainable Development Goals of household’s financial access to UHC. Thus, the study will also serve as a baseline for tracking UHC for household financial access to malaria care that the country has embarked on.

## Introduction

Different countries have put up various mechanisms to achieve UHC for Malaria [1]. Despite progress made in achieving the three dimensions of UHC for Malaria, significant challenges remain in addressing the Global burden of the disease [2]. Globally it is estimated that about 3.2 billion people—nearly half of the world’s population—are at risk of malaria [3, 4]. In 2016, WHO estimates show that 216 million cases and 445,000 deaths of malaria occurred globally. Africa accounted for 90% and 91% of the malaria cases and deaths respectively [5]. The global malaria incidence was estimated at 63/1000 persons at risk of malaria and *plasmodium falciparum* accounted for 99% of the malaria cases in Africa [5]. Funding for malaria for the 41 high burden countries (HBCs) is still below USD2 per person at risk of malaria and 83% (34/41) of the HBCs rely heavily on external funding for malaria control.

Apart from negative health consequences, malaria poses an economic burden both at macro and microeconomic levels [3]. Malaria imposes a financial burden on the poorer and more vulnerable households, particularly in malaria-endemic regions. The impact is diverse and worsened if the illness affects the productive age groups; other than increasing household expenditure, it negatively affects labour supply. Malaria treatment expenses and lost productivity account for a substantial portion of the annual income and savings reduction for poorer households, especially those who depend on agriculture for a living and who may not have other forms of paying for health services such as prepaid health insurance [6]. The economic burden attributable to Malaria remains high in developing countries. Malaria is estimated to cost African countries over $12 billion per year in direct losses and an average loss of 1.3% of economic growth per year. This is despite the fact that it could be controlled for a fraction of this cost [4].

A number of malaria prevention, treatment and control have been implemented worldwide to curb the spread of the disease [7]. These have included but not limited to; Indoor residual spraying (IRS), Intermittent Preventive Treatment (IPT) and use of Artemisinin Combination Therapy (ACT) as first-line therapy which has recently received attention especially with the use of improved diagnosis such as Rapid Diagnostic Tests (RDTs) [3]. An integrated approach that combines preventative measures, such as Long Lasting Insecticide-Treated Nets (LLINs) and Indoor Residual Spraying (IRS), coupled with improved access to effective anti-malarial drugs is the key to addressing the challenge of reducing the burden of malaria [4]. In spite of all the many achievements in malaria interventions, access to prompt and effective case management still remains a challenge during visitation and hospitalization in health facilities in Zambia [7]. Millions of people are still not receiving the services they need to prevent and treat malaria [2].

Chuma et al [8] further notes that access to malaria treatment is much broader than the above-mentioned interventions of IRS, administration of IPT and ACTS, distribution of LLTNs, and use of RDTs. The treatment prevention strategies must go further to consider the demand and supply side factors such as financial and physical access barriers, perceptions of illness causation, and perceived and actual effectiveness of treatment, Provider attitudes, quality of care, drug dosage adherence, the dynamics of power within households, and the availability of high-quality information. At the household level, malaria poses a direct threat due to time and productivity losses, treatment costs, loss of household income, and even premature death. In addition, previous research has demonstrated that the demand for health care among the poor is sensitive to price changes, indicating that any increase in the price of health services may inhibit demand or result in catastrophic expenditures for poor households. Therefore, medical services may be delayed or denied, increasing the risk of natural transmission [9]. Households costs associated with malaria care present important barriers to accessing malaria services especially when these costs are catastrophic for households which may result in delay in seeking care or seeking inadequate care due to financial difficulty [10].

The WHO estimates that households spending 40% or more of their non-food expenditures on treatment are likely to be poor. In this regard, the poor are likely to be the most affected, as they spend a greater proportion of their income on medical care than do wealthier groups. Prior studies on the cost of illness have demonstrated that in low- and middle-income countries, health expenditures frequently exceed 10% of household income. Although costs above this threshold are regarded as potentially catastrophic, there are arguments that any health expenditure that prevents households from meeting their basic needs is catastrophic and may not, in reality, represent high healthcare costs [11]. Further, it has been shown that a large number of households are pushed into poverty on account of spending on health. For example, in 2010, about 122 million or 1.8 percent of the world’s population were impoverished by OOP spending on health, with Africa having the highest incidence of impoverishment [12].

In Zambia, significant strides have been recorded in combatting the burden of malaria leading to the reduction in malaria incidence and mortality [13]. The reduction in malaria incidence and mortality in Zambia has been attributed to factors such as the increase in the use of ITNs, increased coverage with IPT3 & IRS, increased diagnostic testing by blood slide or RDTs in children, and use of ACTs for treatment of malaria [13, 14]. Other interventions have included the training and use of community health malaria agents to test and treat malaria in the communities, as well as the provision of on-site malaria management and treatment training and mentorship. Additionally, improved coordination of malaria control interventions in the country through elevation from a unit to a directorate, improved coordination of donor support, registering of malaria technical working groups in the districts and scheduled quarterly visits to lower levels were registered as being instrumental in improving implementation of malaria interventions [13, 15].

## Methods

### Study design and setting

This study used a nationally representative cross-sectional survey of households in both rural and urban areas of Zambia conducted in 2014. The survey was conducted among household in all the ten (10) provinces of Zambia namely: Central, Copperbelt, Eastern, Luapula, Lusaka, Muchinga, Northern, North-Western, Southern and Western. At the time of the study, Zambia housed about 16 million inhabitants and about 59% of whom were residing in urban areas [16]. The data analysed in this malaria OOP study was collected as part of the larger ZHHEUS conducted nationally.

### Study sample size and sampling strategy

The sample for the ZHHEUS comprised of 12,000 households selected using a two-stage stratified sampling approach. Overall, a 99.4 response rate was achieved. In this paper we focus on a sub sample of 2,005 households that had at least one member suffering from malaria with a recall period of four weeks for out-patients and six months for the in-patient respectively, resulting into a total of 6,348 malaria patients. For each household, the head of the household answered all questions on behalf of other household members. The survey collected data on all malaria cases and costs incurred on all reported cases in the household. The sampling frame included all households residing in Zambia at the time of the survey. Institutionalized population groups and diplomats accredited to Zambia were excluded. The sampling frame consisted of a list of standard enumeration areas [SEAs) for the whole country. The SEAs were as developed from the 2010 Population Census frame. The list has information on the expected number of households and population. There are about 25,000 SEAs countrywide.

The study employed a two-stage stratified cluster sample design. In the first stage, SEAs were selected within each stratum using the probability proportional to estimated size (PPES) procedure. During the second stage, 20 households were selected from each of the 600 sampled SEAs using the systematic random sampling method. This method ensured that each household had an equal chance of being selected.

### Selection of primary sampling units

For each stratum (province, rural/urban), the list of SEAs was ordered by SEA identification numbers. The list included, for each SEA, the total number of households and the cumulated measure of size (by adding the number of households down the list). Then, a sampling interval was calculated by dividing the total number of households (final cumulated measure of size), by the number of sample SEAs allocated to the stratum. In addition, a random number between 1 and the sampling interval was picked as the random start for the systematic PPES selection of SEAs.

### Sample weights

In order for the survey estimates to be representative at national or any domain level, it was necessary to weight the sample data with appropriate expansion factors. Sampling weights were also needed to compensate for unequal selection probabilities, non-coverage, non-response, and for known differences between the sample and the reference population. Thus, sample weights act as boosting factors to represent the number of units in the survey population that are accounted for by the sample unit to which the weight is assigned. These weights were calculated, included in the data set and used to draw inference to the population.

The weight for each sample unit (household) was equaled to the reciprocal/ inverse of its probability of selection. The probability of selecting cluster *i* was calculated as:

$$P_{hi}=\frac{a_{h}M_{hi}}{\sum_{i=1}^{N_{k}}M_{hi}}$$

where: *P*_*hi*_ is the first stage sampling probability of (SEA), a_h_ is the number of SEAs selected in stratum *h*, *M*_*hi*_ is the size (total households listed) of the i^th^ SEA in stratum h, and $\sum_{i=1}^{N_{k}}M_{hi}$ is the total size of stratum h e.g., Central province rural.

The selection probability of the household was calculated as:

$$P_{hij}=\frac{b}{M_{hi}}$$

Where *P*_*hij*_ is the probability of selecting a household in the i^th^ SEA in stratum h, b is the number of households selected in the SEA. The weight or boosting factor for a household is thus, given as:

$$W_{hij}\frac{1}{P_{hi}*P_{hij}}$$

### Post stratification adjustments

The survey collected data on all usual household members. The weighted sum of the total number of household members (household size) is supposed to give a fairly good estimate of the current population in a particular stratum such as province, rural/urban and national level for which this survey was designed.

The weighted results generated underestimated the total population when compared to the CSO projected population. This was mainly due to possible under-coverage of households during listing and the lack of updating of the cartographic frame to reflect population growth over time. The frame was based on 2010 population. This necessitated the adjustment of the base-weights to reflect the 2014 population projections. The procedure for adjusting the weights based on population projections is given below:

$$r=\frac{Y_{proj}}{Y_{est}},$$

Where *r* is the adjustment factor, *Y*_*proj*_ is the Projected Population of the stratum e.g. Lusaka province and *Y*_*est*_ is the estimated population of the domain from the survey.

The final weight was given by:

$$W_{adj}=W_{hji}*r$$

Where *W*_*adj*_ is the adjusted final household weight.

### Estimation process

Let *Y*_*hij*_ be an observation on variable Y for the j^th^ household in the i^th^ SEA of stratum h. Then the estimated Total for stratum h is:

$$y_{h}=\sum_{i=1}^{a_{h}}\sum_{j=1}^{n_{h}}w_{hi}y_{hij}$$

where, *y*_*h*_ is the estimated total for stratum h, *w*_*hi*_ is the weight for the j^th^ household in the i^th^ SEA of stratum h, j = 1-*a*_*h*_ is the number of selected clusters in the stratum, j = 1-*n*_*h*_ is the number of sample households in the stratum.

### Data collection procedures

At each household, the head of the household was the primary respondent. After administering the consent form, individual and household information was collected from the household head covering household characteristics, malaria history, health seeking behavior, access to healthcare and coverage of services, public and private health care utilization, perceived quality of care, health care expenditures, out of pocket spending, willingness to pay for health services and social health insurance. Households were asked questions if they had suffered from malaria. Additionally, households were also asked questions if they incurred any costs on malaria treatment, diagnosis, consultation, transport and drugs. Furthermore, Households were asked if they had incurred any costs on supplementary costs such as bed nets.

### Data analysis

The merged and cleaned data was analyzed in STATA version 13. Frequencies were run for respondent characteristics, and Household OOP payments for malaria were computed using the questions that sought if Households had incurred any OOP payments for malaria using the key variables of expenditure on malaria such as the expenditure related to malaria on consultation, drugs, diagnosis, ITNs, hospitalization and any other costs as related to malaria. The quintiles were calculated using the standard calculation, and the CHEs was calculated using the recommended WHO of an amount spent on health exceeding 10% of total consumption and the results were also calculated using the 40% percent threshold of capacity to pay which the WHO defines as the percentage of health expenditure that exceeds 40% of capacity to pay. Impoverishment was calculated by comparing the poverty headcount, that is the percentage of households that were poor based on household total consumption but before spending on health to the percentage of poor households after household out-of-pocket spending on health. To classify a household as poor, we used a poverty line, a threshold based on a fixed expenditure or consumption level. The poverty line typically specifies the amount of money that is required to meet a minimum standard of living, such as basic nutritional requirements and essential non-food necessities (basic clothing, housing, etc.). At the time of the survey Zambia’s poverty line was estimated at K214 percapita per month. We estimate the incidence of impoverishing spending for the whole sample as well as for each quintile of total expenditure. The measure is based on Wagstaff and van Doorslaer (2003) [17].

## Ethical considerations

Ethical Clearance was granted under the provisions of the Census and Statistics Act number 127 of the Laws of Zambia. No identifying information of individuals was collected in this study. Written informed consent was obtained from all study participants.

## Limitations of the survey

The survey was the first to be conducted of this nature, so indicators may not be compared directly with other indicators that are mostly compiled using other survey such as the Living Conditions and Monitoring Surveys (LCMS). In addition, accuracy of income and expenditure data may have been affected by respondents’ inability to recall exact values.

## Results

### Sample description

Overall, 2,005 households members reported having had a malaria case resulting into 6,348 malaria patients. Majority of the patients were female, rural residents and those aged above five years. About 72% had sought treatment for malaria. Most of the malaria patients sought care at public primary health care level representing about 82% of the respondents and most of the cases were uncomplicated malaria. The rest of the malaria patient characteristics are summarized in Table 1 below.

**Table 1:** Malaria patient characteristics.

Variable	Frequency	Percentage, %
**Gender:**		
Male	2,901	45.70
Female	3,447	54.30
**Setting**		
Rural	4,526	71.30
Urban	1,822	28.70
**Age**		
< / = 5 years	1,496	23.57
>/ 5years	4,852	76.43
**Sought treatment**		
Yes	4,547	71.63
No	1,801	28.37
**Where treatment was sought**		
Public health facility (Third Level)	47	1.03
Public health facility (Second Level)	363	7.98
Public health facility (Primary Level)	3706	81.51
Private health facility (Hospital)	32	0.70
Private health facility (Clinic)	66	1.45
Pharmacy	96	2.11
Traditional healer	194	4.27
Other	43	0.95
**Malaria prevalence**		
Had Malaria	6348	11
**Type of malaria**		
Uncomplicated	4142	91.09
Severe	405	8.91
**Wealth Quintile**		
Poorest	1,519	23.93
Second	1,508	23.76
Third	1,340	21.11
Fourth	1,146	18.05
Richest	835	13.15

Table 2 below indicate that most of the Household OOPs were due to transportation costs. Diagnosis and treatment also represented the major costs incurred by households who sought care due to malaria. Expenditure on ITNs represented the least of the expenditures incurred by Households on malaria. Other expenditures mainly consisted of such costs as the purchase of books used as a medical record at the health facility, and bypass fees incurred by the patients for not following Ministry of Health referral procedures.

**Table 2:** Household out of pocket payments among patients with malaria who sought care.

Source of cost	Average OOP in (ZMW)	Proportion of mean household income
Consultation	7.20	0.12
Diagnosis	29.28	1.44
Treatment (Medicines)	25.36	0.61
Hospitalization	10.46	1.87
Transport	38.02	1.15
ITN	0.00	0.00
Other	12.99	0.24

Figures are computed for all patients who reported malaria regardless of whether they incurred oop payments or not.

All figures are annualized

Table 3 below indicates the distribution and incidence of catastrophic OOP by wealth quintile. The table also shows the households that had payments that were classified as catastrophic in nature out of the total number of households who indicated that they had malaria as shown on the table below.

**Table 3:** Distribution and Incidence of catastrophic OOP by wealth quintile.

	Poorest	Second	Middle	Fourth	Richest	Overall
Mean annual household income (ZMW)	4739.4	5738.9	9378.9	9699.7	43257.5	14134.3
Average capacity to pay* (ZMW)	2290.3	2890.0	5088.9	5651.6	31147.7	5935.7
Subsistence expenditure	2449.3	2840.9	4290.0	4048.1	12109.7	8198.5
Proportion of subsistence expenditure to annual Income	0.5168	0.495	0.458	0.417	0.2799	0.420
Households with CHE (%)						
CHE type A = >10% of household income	194 (27)	175 (24)	161(22)	123 (17)	68 (9)	721 (100)
CHE type B = ≥40% of capacity to pay	169 (29)	160 (27)	128(22)	92 (16)	40 (7)	589 (100)

*Capacity to pay = household income minus subsistence expenditure (income after expenditures for food).

Based on Table 3 above, the results show that average household income increases as households who spend on malaria move from the poorest to the richest quintiles, similarly capacity to pay and subsistence expenditure moves in the same direction. However, the proportion of subsistence expenditure reduces from the poorer households to the richer as the poorer households spend most of their income on food compared to the poorer households. 27% and 29% of the households who faced catastrophic health expenditure where from the poorest households and only 9% and 7% from the richer households measured at both the 10% and 40% thresholds of CHE respectively.

Table 4 indicates the determinants of catastrophic health expenditure for malaria. Based on this table, the odds of CHE was higher among patients who came from households that could be classified as poor. Households that reported hospitalization due to Malaria were several times more likely to incur CHE. Further, the type of health facility is a significant determinant of CHE with the odds being higher for those that sought malaria care in private facilities compared to those that utilized public facilities. Distance from between the area of residence and the health facility visited is a determinant of CHE with patients staying further likely to incur CHE. Households with a larger number of members were more likely to report CHE. Age, sex and region of residence are not significant predictors of CHE.

**Table 4:** Determinants of catastrophic health expenditure for malaria (CHE = 1, Non CHE = 0).

Logistic regression	Number of obs		=	2,005	
	LR chi2(7)		=	90.22	
	Prob > chi2		=	0.00	
Log likelihood = -338.61	Pseudo R2		=	0.1176	
**Variable**	**Odds Ratio**	**Std. Err.**	**P-value**	**[95% Conf. Interval]**	
Age of Household Head in years	1.000	0.007	0.995	[0.986	1.014]
Sex of Household Head (1 = Male, 0 = Female)	1.087	0.242	0.708	[0.703	1.681]
Region of residence (1 = Urban, 0 = Rural)	1.205	0.280	0.423	[0.764	1.898]
Household size	1.161	0.044	0.000	[1.077	1.251]
Education (1 = Formal education, 0 = None)	1.577	0.560	0.200	[0.786	3.165]
Employment status of Household Head (1 = unemployed, 0 = employed)	1.353	0.307	0.183	[0.867	2.111]
Household has child <5 years (1 = Yes, 0 = No)	0.991	0.262	0.972	[0.590	1.663]
Facility type (1 = Private, 0 = Public)	1.264	0.481	0.038	[0.599	2.666]
Hospitalisation (1 = Yes, 0 = No)	6.434	1.890	0.000	[3.618	11.44]
Insurance cover (1 = insured, 0 = uninsured)	1.061	0.576	0.912	[0.367	3.074]
Poverty (1 = Non poor, 0 poor)	0.915	0.260	0.055	[0.524	1.598]
Distance to health facility in KMs	1.038	0.010	0.000	[1.019	1.058]
Constant	0.016	0.023	0.004	[0.001	0.274]

Table 5 shows the percentage of households pushed below the poverty line by out-of-pocket health spending. The estimates show that overall, about 0.6 percent of the households of the total sample dropped into poverty as a result of OOP payment for Malaria health services. The impoverishing effect is more evident among the poorest households where about 1.2 percent were forced into poverty due to OOPs.

**Table 5:** Incidence of impoverishing from health spending.

Category	Poverty line	Incidence (%)	Concentration Index	p-value
*Wealth quintiles*				
Poorest	214	1.245	0.837	0.000
Second	214	0.936	-0.399	0.019
Third	214	0.259	0.170	0.598
Fourth	214	0.078	0.309	0.599
Richest	214	0.310	-0.189	0.498
Full sample	214	0.566	-0.337	0.000

## Discussion

The results of this study indicated that despite the services for malaria being free of charge at public health facilities, household still report significant costs during utilisation of malaria services. The composition of OOP payments was higher for transport, followed by diagnosis and medicines, the lowest was for ITNs payments. The high costs of transport that the households incurred when accessing health services could be due to the long distance that the households have to travel to the health facilities to access malaria services as most of the facilities in Zambia are still outside the 5 km radius [22]. In a study that was done in Malawi in 2012 on household costs among patients hospitalized with malaria, the evidence showed that the total household costs averaged $17.48 per patient [10]. Like the results of this study, household distance from the health facility also proved to be one of the significant drivers of overall costs. At $3.8 (10 ZMW = $1) transport was the major contributor to OOP payments in Zambia for households utilising malaria services. It is not surprising that a similar studies that were done in in Rwanda, Burkina Faso, Chad and Congo- Brazzaville also found that across these countries the indirect costs on average accounted for about 79% of the total cost of treatment which was US$ 6.87 out of total costs of US$ 9.84 [18].

The high expenditure on drugs ($2.54) could be explained by either the drugs running out of stock resulting into patients been given a prescription like was the case in the study that was done in Mozambique in which most patients who sought care ended up with a prescription or patients that had to access the services from private facilities but do not have insurance cover [19]. Hence they had to incur OOP payments at the time of utilising the services. The median financial cost of treating an episode of uncomplicated malaria ranged from $2.36-$23.65 in a systematic review of cost and cost effectiveness of malaria interventions in selected countries [3]. Another study that was done on the economic burden of malaria on the household in south-central Vietnam also indicated that each episode of malaria was estimated to cost the household an average of $11.79 (2005 prices) including direct costs for travel and treatment [20].

It was interesting to note that the results of this study showed expenditure on ITNs that was very low indicating that the country strategy of mass distribution on ITNs is working to give the country universal financial protection on ITNs for malaria. Similarly, in a study that was done in Tanzania, the results showed that every fortnight, households spent an average of less than a dollar (S $0.18) on nets and their treatment [21]. The Malaria Indicator Surveys (MIS) conducted in Zambia between 2006 and 2012 have shown substantial progress in making malaria control services widely available in Zambia including possession and use of ITNs [22]. It is therefore not surprising that other studies have indicated that ITNs should be provided via the public sector as a public good, because malaria is linked to poverty [23].

Although the results of the study indicate that richer households spent more on malaria, the proportion of households that incurred CHEs was higher for those who are in the lower levels of the SES than those in the richest quintiles indicating that poorer households were prone to CHEs despite spending less on malaria. This was the case at both the 10% and 40% thresholds. Similarly, in a cross-sectional household survey that was conducted to estimate the household economic burden of malaria in Zimbabwe, the results showed a huge spread averaging between $3.22 and $56.60 for managing a malaria case. Of all the households that suffered catastrophic expenditures, 12.5% of them had a member of the family hospitalized and 35% suffered catastrophic expenditures using a 40% threshold [6]. Similar studies that have been done in the region also show high CHE on malaria. In the study that was done in the Democratic Republic of Congo (DRC) the results indicated that households facing CHE due to malaria at the two thresholds of 40% and 10%, the incidence reached 81.1%, and 46.4% respectively [24]. This high level of CHE could be attributed to different levels of malaria interventions that country mechanisms have put in place in the region.

In the study that was done in Uganda, the Household direct costs of seeking care from health facilities due to malaria were significantly higher for urban-based caregivers than the rural counterparts [25]. The results of this study has also indicated that the odds of CHE was less in the rural areas than in the urban areas which is good for protecting the poor especially that the country removed user fees at primary level. In published studies, the incidence of poverty due to OOP are varied, mainly due to differences in the methods used to measure healthcare expenditures and the poverty thresholds used to calculate poverty. But what is consistent across the majority of studies, including our own, is that the effects of OOPs on poverty appear to be more pronounced among the poorest households.

## Conclusion

The findings of this study contribute to knowledge on Household Catastrophic Health Expenditure for malaria in Zambia and the OOP payments that the households are incurring when they have to sought care related to malaria. The results are timely as the country has embarked on an ambitious goal towards malaria elimination as one of the key goals in the transformation agenda as they provide a baseline at which UHC for financial protection goal for malaria can be measured. Therefore, it is hoped that having knowledge on the OOP payments and CHE on malaria will enable the country better position itself on how to target interventions towards achieving household financial access for malaria services the disease that pose as a major public health concern in Zambia. There is need to further consider malaria interventions targeting poorer households if the Country is to attain UHC for financial protection for accessing malaria interventions for countries in which malaria is a major public health concern. Further, the government should aim to increase the quality of services in public sector to reduce on the number of poorer populations accessing private health facilities. In addition, the government should continue to investment more in the construction of health facilities stocked with anti-malaria medications that the population should continue to access free of charge. Increasing the number of health facilities will bring closer to the people of Zambia health services which in turn will reduce the high transportation costs incurred by patients and caregivers who reside further away from health public health facilities.
